# Peripheral quantitative computed tomography detects differences at the radius in prepubertal children with cystic fibrosis compared to healthy controls

**DOI:** 10.1371/journal.pone.0191013

**Published:** 2018-01-11

**Authors:** Catherine E. O’Brien, Gulnur Com, John Fowlkes, Xinyu Tang, Laura P. James

**Affiliations:** 1 Department of Pharmacy Practice, University of Arkansas for Medical Sciences College of Pharmacy, Little Rock, Arkansas, United States of America; 2 Department of Pediatrics, University of Southern California, Keck School of Medicine, Los Angeles, California, United States of America; 3 Barnstable Brown Obesity and Diabetes Center University of Kentucky, Lexington, Kentucky, United States of America; 4 Department of Pediatrics, University of Arkansas for Medical Sciences, Little Rock, Arkansas, United States of America; 5 Arkansas Children’s Hospital, Little Rock, Arkansas, United States of America; Universidad Europea de Madrid, SPAIN

## Abstract

**Introduction:**

In 2015, 11.9% of people with cystic fibrosis (CF) in the United States had osteopenia, 5.1% osteoporosis, and 0.3% experienced a fracture. Screening for CF-related bone disease starts in childhood, and dual energy x-ray absorptiometry (DXA) is the recommended method. It is unknown whether peripheral quantitative computed tomography (pQCT) can detect deficits earlier than DXA. This study compared pQCT and DXA scans in a group of pre-pubertal children with CF and healthy controls.

**Methods:**

This was a cross-sectional study of children at Tanner stage 1. A pQCT scan of the radius at proximal and distal sites was performed plus a total body DXA scan. Serum C-reactive protein, interleukin-6 and tumor necrosis factor-alpha were also measured.

**Results:**

A total of 34 subjects completed the study; 14 with CF and 20 controls. At the distal radius, pQCT showed a lower total bone mineral density (BMD) Z-score for the CF group (P = 0.01 and P = 0.03 for 2 different reference databases) compared to controls. At the proximal site, the polar strength-strain index was lower in the CF group (P = 0.017). Finally, the total body BMD Z-score by DXA was lower in the CF group, although it did not meet the definition of reduced bone density (P = 0.004). Biomarkers of inflammation were not different.

**Conclusions:**

In this group of pre-pubertal children with CF, measures of bone strength and density by both pQCT and DXA were reduced compared to healthy controls.

## Introduction

Cystic fibrosis (CF) is a genetic disease that is the result of a mutation in the gene that encodes the cystic fibrosis transmembrane conductance regulator (CFTR). CFTR functions as a chloride channel in the epithelial cells that line the airways and other organ systems, and its dysfunction or absence results in thick and tenacious pulmonary secretions. Although the majority of patients eventually die of respiratory failure, other organ systems are also affected by the disease including the skeleton.[1] Among people with CF in the United States in 2015, 11.9% had osteopenia, 5.1% had osteoporosis, and 0.3% had experienced a fracture.[2] The classifications of osteopenia and osteoporosis were based on screening by dual energy x-ray absorptiometry (DXA). Patients with CF are screened for bone disease with DXA and this starts in childhood according to guidelines from the CF Foundation.[1] Screening with peripheral quantitative computed tomography (pQCT) is not currently recommended as there is insufficient data to evaluate its use, especially in children. However, pQCT provides measurements of parameters that DXA scans do not. It provides bone mineral density (BMD) measurements for trabecular and cortical bone separately, can measure cross-sectional area, cortical thickness, and can provide a calculated strength-strain index. These additional parameters may potentially provide greater insight into early changes in CF-related bone disease. A study published in 2009 compared bone studies in young adults with CF using DXA and pQCT and found deficits by pQCT but not DXA, most notably reduced cortical thickness at the radius.[3] A more recent study evaluated pQCT parameters at the non-dominant tibia and radius in children and adolescents with CF and found no deficits prior to puberty.[4] There is growing interest in the use of pQCT to evaluate skeletal health in people with CF, but still there is little data available evaluating its use, especially in younger children with CF. However, if screening with pQCT is able to detect deficits earlier than with DXA, earlier detection of bone health abnormalities could lead to more rapid treatment to mitigate the clinical impact of altered bone integrity, such as fracture.

The primary goal of this pilot study was to compare pQCT Z-scores at the radius between children with CF and their healthy peers. Secondary aims included a comparison of total body bone mineral density (BMD) Z-scores as measured by DXA, and a comparison of biomarkers of inflammation between the two groups.

## Methods

This was a single-center cross-sectional pilot study comparing total body DXA and pQCT of the non-dominant radius between pre-pubertal children with CF and healthy, age-matched peers. Children 6–12 years of age and of Tanner stage 1 development were eligible for inclusion. The study was approved by the Institutional Review Board (IRB) and State Medical Board. Written informed consent was obtained from a legal guardian, and written assent was also obtained for children 7 years of age or older. The consent procedure was approved by the IRB.

Exclusion criteria included a BMI less than the 3^rd^ percentile or greater than the 95^th^ percentile, fracture within the past 6 months, lung transplantation, current pulmonary exacerbation or infection, bisphosphonate or growth hormone treatment within the past 5 years, glucocorticoid therapy via oral inhalation, oral liquid or tablets, or intravenous administration within the past 6 months, FEV1 less than 40% of predicted for children with CF, or concomitant disease known to cause bone disease.

Study participants presented for a single study visit during which height and weight were obtained, blood was drawn, and DXA and pQCT scans were performed. Bone age was determined by an x-ray of the non-dominant hand as previously described by Greulich and Pyle.[5] Blood based markers of inflammation, including TNF-alpha and IL-6, were quantified by commercially available ELISA kits from Quantikine using methods recommended by the manufacturer. C-reactive protein was measured by the hospital clinical laboratory using standard methods. BMI percentiles were determined using the online Centers for Disease Control (CDC) Child and Teen BMI percentile calculator, which is based on CDC growth charts.[6]

All DXA scans were performed with the Hologic QDR 4500A bone densitometer (Hologic, Inc., Beford, MA), and total body less head (TBLH) BMD was given as a Z-score and in total grams. The pQCT was performed with the Stratec XCT 2000 (Stratec, Inc., Pforzheim, Germany) and scans were performed at the 4% of the length of the forearm (distal) and 66% of the length of the forearm (proximal) sites of the non-dominant radius after a scout view to identify the growth plate. The scans were performed at a speed of 25 mm/sec with a voxel size of 0.4 x 0.4 x 2 mm. The forearm was positioned in an arm rest with bubble wrap between the arm rest and each child’s arm to provide non-echogenic space on the scan between the arm rest and the arm. They were encouraged to remain as still as possible during the scan. The following parameters were determined: total and trabecular BMD for the 4% site; and cortex width, cortical area, cortical cross sectional area, total BMD, and cortical BMD for the 66% site. Z-scores were calculated using the reference databases developed by Rauch et al[7,8] and Ashby et al.[9] Bone age was used when calculating Z-scores to correct for possible differences in body size between the groups. The polar strength-strain index (SSI) was calculated as a measure of torsional bone strength at the 66% site. The axial SSI was not used as it can be affected by how the arm is positioned during the scan. The total dose of radiation for the pQCT scan, including the scout view, was 6 mrem.

As this was a pilot study and there was no preliminary data available, there was not a power calculation. The goal was to recruit a convenience sample of participants. Summary statistics are expressed as median (IQR) for continuous variables and frequency for categorical variables. Comparisons between the two groups for continuous variables were performed using Wilcoxon rank-sum test. The comparisons between groups for categorical variables were performed using chi-square or Fisher Exact test as appropriate. The data were analyzed using statistical software R v.3.2.3 (R development Core Team, Vienna, Austria).

## Results

A total of 39 study subjects met inclusion criteria and participated in the study, 15 with CF and 24 healthy controls. Several subjects were excluded due to motion artifacts in the pQCT scans; 1 from the CF group and 4 controls had motion artifacts at both the distal and proximal sites, and data from these subjects was not analyzed. One subject from the CF group had a motion artifact at the proximal site only. Data from this subject was included in the baseline characteristics and pQCT results for the distal site scan. The groups were comparable by age and BMI percentile (Table 1). Notably, the median BMI percentile in the CF group was at the goal of at least the 50^th^ percentile. Lung function in the CF group was also excellent, with a median FEV1% predicted of 100.0 (88.5, 117.8). Bone age was comparable in the CF group, 10.0 (6.8, 11.0) years; and in the control group, 10.0 (8.8, 11.0) years (p = 0.39). No differences were detected in serum concentrations of IL-6, TNF-alpha, and CRP in the two study groups.

**Table 1:** Characteristics of study participants.

	CF	Controls	P
	N = 14	N = 20	
Age in years Median (IQR)	10.1 (8.0, 10.9)	9.6 (8.5, 11.0)	0.99
Gender, N (%)			0.54
F	4 (28.6)	9 (45.0)	
M	10 (66.7)	12 (50.0)	
Race / Ethnicity, N (%)			0.85
White	11 (78.6)	14 (70.0)	
Black	1 (7.1)	2 (10.0)	
Hispanic	1 (7.1)	2 (10.0)	
Other	1 (7.1)	2 (10.0)	
CFTR Genotype, N (%)		NA	NA
*Phe508del* homozygous	7 (50.0)		
*Phe508del* heterozygous	5 (35.7)		
Other	1 (7.1)		
^a^FEV1% of predicted, median (IQR)	100.0 (88.5, 117.8)	NA	NA
BMI, median (IQR)	16.6 (16.0, 17.7)	17.1 (16.0, 18.4)	0.53
BMI percentile Median (IQR)	56.2 (46.4, 67.0)	60.5 (42.3, 82.6)	0.58
IL-6, pg/mL	1.60 (1.10, 2.22)	1.09 (0.66, 2.30)	0.33
TNF-alpha, pg/mL	3.63 (1.76, 5.28)	2.26 (0.70, 3.89)	0.18
CRP		)	0.19
< 5 mg/L	10 (71.4)	18 (90.0)	
5–10 mg/L	2 (14.3)	2 (10.0)	
Missing	2 (14.3)	0 (0)	

Abbreviations: CF–cystic fibrosis, CFTR–cystic fibrosis transmembrane conductance regulator, FEV1 –forced expiratory volume in 1 second, BMI–body mass index.

^a^N = 11 for FEV1.

At the distal site of the radius, total BMD and total BMD Z-scores by pQCT were lower in the CF group than the control group (Table 2). This finding was observed regardless of the reference database used to calculate the Z-scores. No differences were noted between the trabecular BMD or trabecular BMD Z-scores by pQCT at the distal site. No differences were noted from pQCT scans in the cortical area, cross sectional area, cortical BMD, total BMD, or Z-scores at the proximal site (Table 3). However, torsional bone strength, as measured by the polar SSI, was significantly lower in the CF group compared to the controls.

**Table 2:** pQCT results at distal site.

	CF	Controls	P
	N = 14	N = 20	
**pQCT at distal (4%) site**			
Total BMD (mg/cm^3^)	304.8 (299.6, 325.6)	349.2 (301.6, 372.6)	0.046
Total BMD Z-score (Ashby)	-0.24 (-0.42, 0.43)	0.97 (-0.14, 1.32)	0.019
Total BMD Z-score (Rauch)	0.59 (0.21, 1.11)	1.72 (0.61, 2.21)	0.018
Trabecular BMD(mg/cm^3^)	201.3 (185.9, 218.4)	213.4 (183.6, 233.5)	0.420
Trabecular BMD Z-score (Ashby)	1.07 (0.45, 1.35)	1.29 (0.11, 1.68)	0.560
Trabecular BMD Z-score (Rauch)	0.36 (-0.48, 0.76)	0.55 (-0.62, 1.20)	0.500

**Table 3:** pQCT results at proximal site.

	CF	Controls	P
	N = 13	N = 20	
**pQCT at proximal (66%) site**			
Total BMD (mg/cm^3^)	656.8 (604.5, 688.5)	596.1 (541.1, 643.3)	0.194
Total BMD Z-score (Rauch)	0.69 (-0.34, 1.63)	0.09 (-0.80, 0.70)	0.224
Cortical BMD (mg/cm^3^)	960.9 (943.7, 1007.9)	1004.6 (969.2, 1017.5)	0.231
Cortical BMD Z-score (Rauch)	-0.52 (-1.39, 0.09)	-0.35 (-0.87, 0.47)	0.552
Cortex width (mm)	1.49 (1.23, 1.70)	1.55 (1.31, 1.74)	0.726
Cortex area (mm^2^)	33.6 (25.9, 45.3)	40.8 (35.0, 48.6)	0.167
Cortex area Z-score (Rauch)	-1.30 (-3.26, 0.01)	-0.82 (-1.58, 0.27)	0.272
Cross sectional area (mm^2^)	66.2 (53.0, 76.6)	81.9 (66.4, 91.1)	0.074
Cross sectional area Z-score (Rauch)	-1.77 (-3.80, -0.36)	-0.67 (-1.09, 0.09)	0.195
Polar Strength-Strain Index	112.3 (95.4, 133.8)	149.0 (123.5, 173.7)	0.017
Polar Strength-Strain Index (Rauch)	-1.26 (-1.82, 0.08)	-0.03 (-0.63, 0.34)	0.106

Although the median total body Z-scores for the DXA scan do not indicate reduced bone density in either group, there was a statistically significant difference in the total body BMD Z-scores between the CF group, -0.70 (-1.30, 0.50); and the control group, 0.35 (-0.25, 1.80); p = 0.01.

## Discussion

In this group of pre-pubertal well-nourished children with CF and good lung function, there were deficits by pQCT compared to the control group including a lower total BMD at the distal radius site, and lower bone torsional strength as assessed by the polar SSI. The total body DXA Z-score was also significantly lower in the CF group compared to the controls, although the median Z-score did not meet the definition for reduced bone density in either group.[10] Multiple studies have shown correlations between BMD with clinical factors including lung function, nutritional status, biomarkers of inflammation, and frequency of antibiotic courses.[11–18] Although we did not collect the frequency of antibiotic courses in this group of children with CF, lung function and nutritional status were good, and there was not a significant difference in the serum biomarkers of inflammation CRP, IL-6, and TNF-a between the CF group and healthy controls. This suggests that there may be an effect of CF disease on bone even in relatively healthy children with CF. Studies evaluating the relationship between biomarkers of inflammation and bone in CF patients have been mixed, although in general have demonstrated an inverse relationship between inflammation and BMD, and a direct correlation between inflammation and markers of bone resorption.[15,17,19]

DXA and pQCT scans measure different things. The DXA scan measures aerial BMD, a 2-dimensional measurement, and the accuracy of results depends on bone size as well as surrounding soft tissue. A smaller skeleton can result in lower DXA values even if the BMD is normal. It also cannot differentiate between cortical and trabecular bone. In contrast, pQCT is a 3-dimensional measurement that is able to measure total BMD as well as the BMD for cortical and trabecular bone separately.[20]

A study by Brookes et al described pQCT scans in children with CF.[4] This study included pubertal as well as pre-pubertal children. Prior to puberty, the study showed no deficits that would impact bone strength. However, in children with CF at puberty, the pQCT parameters started to indicate a compromised bone structure with a decreased bone strength compared to healthy peers. Most of the studies evaluating pQCT parameters in patients with CF have been conducted in adolescents and young adults. Studies utilizing high resolution pQCT (HR-pQCT) have demonstrated compromised trabecular microarchitecture at both the radius and tibia in young adults with CF.[21,22] This translated into reduced estimated bone strength. In a group of children and adolescents with CF, HR-pQCT results demonstrated minimal abnormalities in bone architecture but no severe deficits.[23] An earlier study evaluated DXA and pQCT results of adolescents and young adults with CF. The results demonstrated normal bone mineral density by total body DXA, but decreased cortical thickness of the radius as measured by pQCT.[3] A more recent study by Stahl et al demonstrated that several pQCT parameters, including SSI and trabecular BMD at the distal radius, are able to differentiate between CF patients with 1 fracture or less and those with more than one fracture.[24] Finally, a study by Bai et al compared pQCT results at the radius in children ages 7–18 years with CF with healthy controls.[25] They found that although the children with CF had thicker cortical bone, they still had reduced bone strength compared to controls. This group of children with CF was not limited to Tanner stage 1.

Although children with CF in this study have skeletal deficits as measured by pQCT compared to their healthy peers, there is currently not enough data to support using pQCT over DXA scans for screening purposes in this population. As patients with CF continue to live longer, it becomes increasingly important to understand the pathophysiology of bone disease in this population in order to develop evidence-based screening and treatment protocols that can mitigate fracture risk. A better understanding of early skeletal derangements would improve screening protocols and allow for earlier intervention. More importantly, it may lead to interventions that better fit the pathophysiology of bone disease in this population.

There are several limitations to this study. The primary limitation is the small sample size. Further, as a single-center pilot study, our results may not be applicable to other CF centers. The BMI percentile range for this cohort of CF patients indicates that they are fairly well-nourished, with the median BMI percentile meeting the goal BMI of at least the 50^th^ percentile set by the CF Foundation.[2] The results may be different for CF patients that are undernourished and below this goal BMI. Another limitation is the lack of a robust assessment of physical activity levels. It is possible that the difference in bone density between the CF and controls groups is due to differences in physical activity levels. A limitation related to the pQCT procedure itself is the requirement that the patient be still during the scan. There were several subjects in this study that were excluded due to motion artifacts. This may limit the utility of this method in young children. Finally, the total dose of radiation, 6 mrem, is higher than that of a DXA scan (0.021 mrem) and comparable to approximately one week’s environmental exposure in someone at this study location and with exposure to television and a computer. In determining the appropriate use of pQCT in a population routinely exposed to radiation from other sources, such as chest X-rays, this would also need to be considered when determining the most judicious use of this method in this population.

## Conclusion

This group of children with CF had lower bone density and torsional bone strength compared to their healthy peers in spite of their young age, healthy BMI, and good lung function. Both the pQCT at the radius and the total body DXA scan were able to detect early differences in bone density between these pre-pubertal children with CF and their healthy peers, even within a normal Z-score range. Thus, a high level of vigilance is warranted when trying to maximize peak bone density in children with CF. There is currently not enough data to warrant screening for bone disease by pQCT instead of DXA. These results are similar to other studies that demonstrated some deficits and differences in bone via pQCT scans in children with CF compared to healthy controls.

## Supporting information

S1 Table Study data. (XLSX)
